# The Emerging Roles of Protein Interactions with O-GlcNAc Cycling Enzymes in Cancer

**DOI:** 10.3390/cancers14205135

**Published:** 2022-10-20

**Authors:** Chia-Wei Hu, Jinshan Xie, Jiaoyang Jiang

**Affiliations:** Pharmaceutical Sciences Division, School of Pharmacy, University of Wisconsin-Madison, Madison, WI 53705, USA

**Keywords:** O-GlcNAcylation, O-GlcNAc transferase (OGT), O-GlcNAcase (OGA), cancer, protein–protein interaction (PPI)

## Abstract

**Simple Summary:**

Dynamic O-GlcNAc modification regulates the functions of proteins in a broad range of cellular processes. Dysregulation of the sole enzymes responsible for O-GlcNAc cycling, O-GlcNAc transferase (OGT) and O-GlcNAcase (OGA), and the associated cellular O-GlcNAc profile is a common feature across nearly every cancer type. Here, we highlight recent studies on the structural features of OGT and OGA, as well as the emerging roles and molecular mechanisms of their aberrant protein–protein interactions (PPIs) in rewiring cancer networks, to help identify key protein contacts and functional modules that drive malignancies and to promote cancer therapeutic innovations.

**Abstract:**

The dynamic O-GlcNAc modification of intracellular proteins is an important nutrient sensor for integrating metabolic signals into vast networks of highly coordinated cellular activities. Dysregulation of the sole enzymes responsible for O-GlcNAc cycling, O-GlcNAc transferase (OGT) and O-GlcNAcase (OGA), and the associated cellular O-GlcNAc profile is a common feature across nearly every cancer type. Many studies have investigated the effects of aberrant OGT/OGA expression on global O-GlcNAcylation activity in cancer cells. However, recent studies have begun to elucidate the roles of protein–protein interactions (PPIs), potentially through regions outside of the immediate catalytic site of OGT/OGA, that regulate greater protein networks to facilitate substrate-specific modification, protein translocalization, and the assembly of larger biomolecular complexes. Perturbation of OGT/OGA PPI networks makes profound changes in the cell and may directly contribute to cancer malignancies. Herein, we highlight recent studies on the structural features of OGT and OGA, as well as the emerging roles and molecular mechanisms of their aberrant PPIs in rewiring cancer networks. By integrating complementary approaches, the research in this area will aid in the identification of key protein contacts and functional modules derived from OGT/OGA that drive oncogenesis and will illuminate new directions for anti-cancer drug development.

## 1. Introduction

**Figure 1:**
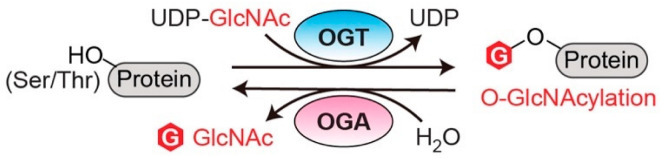
O-GlcNAc cycling enzymes (OGT and OGA) catalyze the reversible protein O-GlcNAcylation. OGT: O-GlcNAc transferase. OGA: O-GlcNAcase. UDP-GlcNAc: uridine diphosphate *N*-acetylglucosamine.

O-linked *N*-acetylglucosaminylation (O-GlcNAcylation) is an essential post-translational modification (PTM) that dynamically regulates numerous protein functions in response to nutrients and stress [1]. Interestingly, only a single pair of human enzymes maintains the homeostasis of this modification: O-GlcNAc transferase (OGT) and O-GlcNAcase (OGA) [2,3,4,5,6]. OGT transfers the GlcNAc moiety from the sugar donor UDP-GlcNAc to the serine or threonine residues of protein substrates (Figure 1). On the contrary, OGA removes the sugar moiety from O-GlcNAcylated substrates (Figure 1). This reversible O-GlcNAc cycle dynamically modulates protein stability, enzymatic activity, protein–protein interactions (PPIs), and the crosstalk with other types of PTMs [7,8]. To date, thousands of O-GlcNAcylated proteins have been identified and they play important roles in remarkably diverse cellular processes, including transcription, translation, apoptosis, cell cycle, protein transportation, mitochondrial function, and signal transduction [7,9,10]. Notably, dysregulation of OGT, OGA, and the associated cellular O-GlcNAc profile is commonly detected in all cancers [11]. For instance, upregulated OGT and O-GlcNAcylation are intimately associated with nearly every cancer-related phenotype, ranging from cell proliferation, epithelial–mesenchymal transformation (EMT), angiogenesis, to metastasis [11,12,13,14]. Emerging evidence also shows that OGT is involved in regulating/activating cancer stem cell potential and resistance in anti-cancer treatments [14,15,16]. On the other side, both up- and down-regulation of OGA protein levels have been observed in different types and grades of cancer [17,18,19,20]. Elevated activity of OGA was also detected in cancer [21]. Furthermore, anti-cancer drugs combined with OGA inhibition using small molecule or genetic approaches have shown synergic inhibitory effects on tumor progression [22,23,24]. More interestingly, a significant correlation between the expression levels of OGT/OGA and the grade/stage of tumors or prognosis has been discovered, promoting mechanistic investigations of these enzymes in cancer [17,25,26]. In general, the abnormal functions of OGT/OGA can make profound impacts on many biological processes, such as metabolic reprogramming, transcription/epigenetic regulation, inflammation, and stress response [27,28,29,30,31]. These dysregulations, often amplified through a large repertoire of O-GlcNAcylated proteins, fuel cancer malignancies and accelerate disease deterioration. These findings raised significant interest in targeting O-GlcNAc cycling enzymes (OGT and OGA) as a potential new anti-cancer strategy. In the past decade, genetic perturbation and the active-site inhibitors of these two enzymes have been widely used to gain fundamental understanding of their roles in normal and disease conditions, and to evaluate their potential for therapeutic development. Exciting progress has been made; however, significant challenges have also become apparent. One of the main challenges is that OGT and OGA are essential enzymes; prolonged knockdown or knockout of either of them leads to embryonic lethality or deterioration of organ functions [32,33]. Inhibition of OGT/OGA’s catalytic site brings similar concerns about unpredictable side effects due to the perturbation of global O-GlcNAcylation [34,35,36,37]. In addition, the non-catalytic functions of OGT and OGA have been recently reported to regulate cell proliferation and tumor cell growth, respectively, indicating that their active-site inhibition may not be sufficient to halt cancers derived from the aberrant non-catalytic functions of O-GlcNAc cycling enzymes [18,38]. Hence, there is a critical need to explore new strategies to target OGT/OGA. To develop such new strategies, a better understanding of how OGT and OGA interact with other proteins (e.g., substrates or non-substrate partners) through regions outside of their immediate catalytic sites would be essential. This knowledge will not only aid in defining the malfunctions of OGT/OGA in complex diseases such as cancer, but also facilitate the development of novel strategies to manipulate the interactions of these enzymes and a subset of proteins without global O-GlcNAc perturbation-induced side effects.

Perturbed PPIs in cancer (cancer-specific PPIs) is one of the key factors in cancer development [39]. Mapping PPIs has provided invaluable insights into the pathophysiological mechanisms in multiple types of cancer [40]. Moreover, aberrant PPIs are arising as new targets for the development of novel cancer therapy. As many PPI inhibitors have entered clinical trials or applications, this has become an important strategy to impede malignant cancer programming with minimal toxicity [41]. Given the manifold functions of O-GlcNAc cycling enzymes, deciphering their roles from a PPI perspective promises fruitful discoveries and may open new doors for cancer therapeutic interventions. Compared to many other cancer-related proteins (e.g., BCL2, p53, etc.), the protein interactions of OGT/OGA have been significantly less explored, potentially restricted by their transient protein interactions with many O-GlcNAcylated substrates and a lack of a conserved recognition motif [42]. However, our knowledge about the OGT/OGA binding partners and their derived PPIs have been steadily advanced in the past few years thanks to the remarkable progress made through structural, proteomic, and biochemical studies. Based on the analysis of 32 different types of cancer using Protein Interaction Network Analysis (PINA, v3.0, Cancer Context tool with mRNA database) [43], an average of over 100 OGT– and 25 OGA–protein interactors have been predicted to be cancer-related, and approximately 16% of them are potential biomarkers for clinical prognosis. Another study of PPI networks predicted OGT as a key cancer “hub” gene making extensive interactions with other proteins [44]. These analyses imply a strong connection between OGT/OGA PPIs and cancer. Here, we review recent discoveries on the protein interactions of OGT/OGA, including their isoforms, from three different perspectives. First, an overview of the structural features of OGT and OGA that are known or have the potential to enable protein interactions with selectivity and flexibility. Second, systematic analyses of OGT/OGA PPIs in cancer models. Third, representative examples of OGT/OGA rewired PPIs that drive cancer malfunctions. These studies not only support the idea of targeting aberrant PPIs of OGT/OGA as a novel, potentially more specific anti-cancer strategy, but also highlight that a better understanding of the oncogenic PPIs derived from OGT/OGA at the molecular and systems levels is much needed for such new developments.

## 2. Structural Insights of O-GlcNAc Cycling Enzymes as Potential Multi-Interface Hubs for Regulating Complex PPI Networks

Analyses using interdisciplinary approaches, including structure, bioinformatics, and multi-omics, have greatly accelerated our understanding of cancer-specific PPI interface properties and topological features. For example, intrinsically disordered regions (IDRs), which play a pivotal role in modulating the plasticity of PPI networks, were found to be significantly enriched in cancer-specific PPIs in the human proteome [45]. Interestingly, a recent study found that protein hubs in cancer-specific PPIs tend to possess more distinct binding sites for various protein partners than non-cancer related proteins [46]. These findings indicate that cancer-specific hubs may have acquired unique structural features to coordinate diverse modules for maintaining the high plasticity and complexity of cancer networks. Of particular interest here, OGT and OGA are potential multi-interface hubs in PPIs, in agreement with their capability to accommodate remarkably diverse protein substrates and the fact that O-GlcNAcylation is often detected in the disordered regions of proteins [47]. While still far from a complete understanding of the protein recognition mechanisms of OGT/OGA, the structural features discussed below start to reveal the molecular basis underlying the selectivity and plasticity of their protein interactions, supporting that the O-GlcNAc cycling enzymes are essential regulators of the dynamic, scale-free PPI networks in cancer.

### 2.1. O-GlcNAc Transferase (OGT)

**Figure 2:**
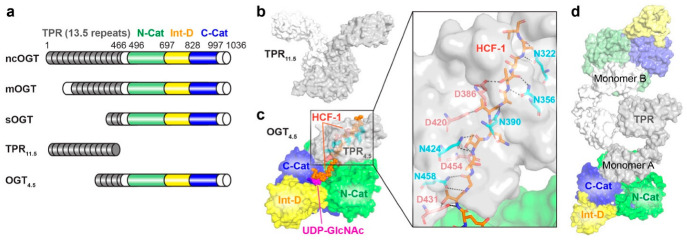
OGT structures provide insights into substrate recognition and protein interactions. (**a**) Schematic diagram of three OGT isoforms (ncOGT, mOGT, and sOGT) and the two truncated OGT constructs for crystallization (TPR_11.5_ and OGT_4.5_). The tetratricopeptide repeat domain (TPR), N-terminal catalytic domain (N-Cat), intervening domain (Int-D), and C-terminal catalytic domain (C-Cat) are colored in gray, green, yellow, and blue, respectively. (**b**) The crystal structure of TPR_11.5_ in a dimer (PDB: 1W3B). (**c**) The crystal structure of OGT_4.5_ in complex with UDP-GlcNAc and HCF-1 peptide (PDB: 4N3C). UDP-GlcNAc and HCF-1 peptide are highlighted in magenta and orange, while the asparagine and aspartate ladders are shown in cyan and salmon, respectively. Domains are colored as in (**a**). Insert: zoom-in at TPR_4.5_ region showing detailed interactions between the peptide backbone/side-chains and asparagine/aspartate residues. (**d**) The cryo-EM structure of OGT in a dimer (PDB: 7NTF). Monomer A (in dark color) is missing two TPRs and monomer B (in light color) is missing five TPRs at the N-terminus.

OGT is encoded by a single gene located on the X chromosome. The human OGT protein contains an N-terminal tetratricopeptide repeat (TPR) domain and a C-terminal catalytic domain, which is split into two halves (N-Cat and C-Cat) by an intervening domain (Int-D) (Figure 2a). Three main splicing isoforms of OGT have been identified, which vary in the length of their TPRs (Figure 2a). The primary isoform of OGT in the nucleus and cytoplasm (nucleocytoplasmic OGT or ncOGT) contains 13.5 TPRs. The isoform in mitochondria (mitochondrial OGT or mOGT) has 9 TPRs [48,49]. The short isoform (sOGT) contains only 2.5 TPRs and is mainly located in the cytosol [48,49]. TPR is a structural motif found in a wide variety of proteins, serving as a scaffold for PPIs [50]. TPR-containing proteins typically possess multiple binding sites enabling distinct interactions with diverse protein ligands that usually do not share a secondary structure or sequence similarity [50]. Interestingly, OGT is the only glycosyltransferase that contains TPR, which has been proposed to serve as a main regulatory region for OGT–protein interactions [51,52,53,54]. This is supported by in vitro studies showing that the N-terminal TPR domain is essential for OGT binding to different protein substrates, including Tet1, Tet2, RNA polymerase II, TARK1, mSin3A, and OGA (the sole enzyme for O-GlcNAc hydrolysis) [55,56]. In addition, the crystal structure of a truncated TPR domain (TPR_11.5_, PDB: 1W3B) revealed that OGT TPR folds into an elongated right-handed superhelix with each repeat displaying a helix–turn–helix motif (Figure 2b) [52]. The extended surface of TPR superhelix offers potential binding sites to various protein partners, contributing to the multi-functionality of OGT in cells. In 2011, the first structure of human OGT containing 4.5 TPRs along with the catalytic and intervening domains (OGT_4.5_, PDB: 3PE3, Figure 2a,c) was reported in complex with UDP [57]. This structure showed that OGT’s catalytic domain contains two Rossmann folds, supporting that it belongs to the glycosyltransferase B (GT-B) family of enzymes [51,54]. Importantly, this crystallizable construct offered a template to obtain a series of ternary complex structures of OGT_4.5_ with UDP-GlcNAc and distinct peptide substrates (for example, OGT_4.5_ complexed with UDP-GlcNAc and HCF-1 peptide substrate in Figure 2c, PDB: 4N3C), providing key insights into the substrate recognition of OGT [58]. In a typical binding mode, a peptide substrate is bound on top of UDP-GlcNAc in OGT’s catalytic pocket and may further extend into the TPR region to form peptide backbone interactions with a ladder of asparagine residues lining inside of the TPR lumen (Figure 2c). This binding mode provides an explanation for OGT’s capability to accommodate a wide variety of peptide sequences. This is also in agreement with the observations of many proteins O-GlcNAc modified in their IDRs as mentioned above [47]. In line with this, mutations of the TPR asparagine ladder impeded global O-GlcNAcylation in cervical cancer HeLa S3 cells, suggesting that TPR is a critical structural element contributing to the diversity and plasticity of OGT–protein interactions [59]. Intriguingly, emerging evidence suggests that the TPR can also provide selectivity towards certain protein substrates. A glycosylation profiling study uncovered an aspartate ladder near the C-terminal part of TPR (Figure 2c), which makes side-chain interactions with the binding peptides, favoring substrates containing positively charged residues in proximity to their O-GlcNAcylation sites [60]. Two recent studies using different methods investigated the asparagine and aspartate ladders spanning the entire TPR lumen and revealed that the asparagine and aspartate residues in different regions of TPR demonstrated differential effects on the O-GlcNAcylation of distinct protein substrates, including OGA and TAB1 [61,62]. This suggests that at least some protein substrates require interactions with an extended TPR surface for efficient O-GlcNAcylation. In contrast, CARM1 and CAMKIV represent a subset of OGT substrates with minimal and partial TPR interaction requirements, respectively [61]. These studies, along with other advances in the field, have begun to reveal that OGT adopts different binding modes via its TPR to interact with distinct protein substrates (or non-substrate partners), which may offer an opportunity to modulate OGT functions toward a subset of proteins without perturbing global O-GlcNAcylation.

Another notable feature of OGT is the Int-D domain that is highly conserved in vertebrate OGTs, but not found in any other glycosyltransferases [3,63]. Interestingly, this domain adopts a unique structural fold with over a dozen of conserved lysine and arginine residues forming a positively charged surface [57]. While the functions of this domain remain elusive, the evolutionary conservation indicates that the Int-D may facilitate OGT interactions with negatively charged ligands, membrane components, or protein surfaces [54,64]. Future studies are expected to define the functional roles of this cryptic domain and may reveal Int-D enabled specific PPIs of OGT. More recently, cryogenic electronic microscopy (cryo-EM) was applied to determine the structure of full-length OGT (Figure 2d) [65]. This structure resembles many features of previously reported crystal structures of TPR_11.5_ and OGT_4.5_, with a slight conformational change detected at TPR10. While the new structure did not provide a high-resolution view at the atomic level, possibly due to the flexibility of the TPR region, it demonstrated that OGT is dimerized with the hydrophobic interaction at TPR 6-7 between the two monomers. This is consistent with the crystal structure of TPR_11.5_ (Figure 2b). Interestingly, the dimerization was shown to be essential for the glycosyltransferase activity of OGT. Disrupting OGT’s dimeric state by double mutation W208A/I211A or single mutation L209A strikingly reduced the catalytic activity of OGT towards the protein substrate TAB1 but not HCF-1 peptide. This is in line with previous reports for other dimeric proteins, suggesting that self-association of OGT may enhance its accessibility and diversity for accommodating different protein substrates and partners [66]. In general, the full-length OGT structure will serve as an important entry point to characterize OGT–protein complex structures in the future. New studies in this direction are expected to provide a better understanding of OGT–protein interactions at the molecular level, further supporting that OGT acts as a multifaceted hub in the PPI networks of biology and disease, such as cancer.

### 2.2. O-GlcNAcase (OGA)

**Figure 3:**
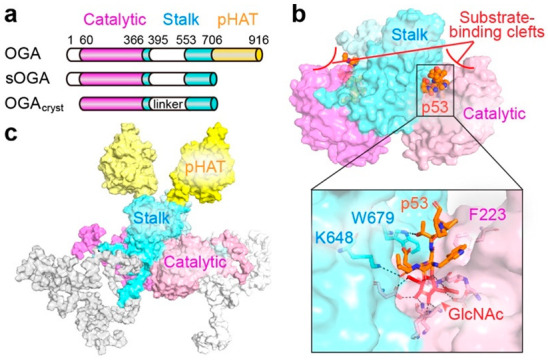
OGA structures provide clues to substrate recognition and protein interactions. (**a**) Schematic diagram of two OGA isoforms (OGA and sOGA) and OGA_cryst_, a reported crystallizable OGA construct. The catalytic domain, stalk domain, and pseudo histone acetyltransferase (pHAT) domain are shown in magenta, cyan, and yellow, respectively. Intrinsically disordered regions of OGA are shown in white. (**b**) The crystal structure of O-GlcNAcylated p53 peptide (highlighted in orange) bound in the substrate-binding clefts of OGA_cryst_ dimer (PDB: 5UN8). Insert highlighted the p53 peptide interactions with OGA residues. (**c**) Predicted full-length OGA model with its domains colored as in (**a**).

OGA is the sole enzyme that can hydrolyze the O-GlcNAc modification from various glycoprotein substrates [4,5]. The unique nucleocytoplasmic localization and optimal activity at neutral pH distinguishes OGA from other acidic hexosaminidases in lysosome [55]. Human OGA is encoded by a single gene on chromosome 10 and can be spliced into two main isoforms, full-length OGA (OGA) and short OGA (sOGA) (Figure 3a) [4,5]. Full-length OGA contains three domains, consisting of an N-terminal catalytic domain showing high sequence similarity to glycoside hydrolase family 84 (GH84) enzymes, a stalk domain, and a C-terminal pseudo histone acetyltransferase (pHAT) domain that is missing in sOGA (Figure 3a) [5,67]. Currently, the full-length OGA structure is not available, but the crystal structures of truncated OGA covering the catalytic domain and most of the stalk domain have been reported by three independent groups using slightly different constructs (OGA_cryst_ in Figure 3a is an example), representing the first models for the eukaryotic GH84 family of enzymes [68,69,70]. These structures show that the catalytic domain of OGA folds into a (β/α)_8_ barrel for GlcNAc binding and hydrolysis, while the stalk domain folds into a helix bundle (Figure 3b). Unlike previously reported bacterial homologs [71,72,73,74], these OGA constructs, as well as the full-length human OGA, are tightly associated dimers, which have been validated by biochemical and biophysical methods [69,70]. The unique human OGA dimer is stabilized by the extensive interactions at the dimerization interface and is important for enzyme stability and catalytic activity [69].

How OGA recognizes its diverse protein substrates has been a long-standing question. It is debatable whether OGA can interact with glycoprotein substrates beyond the O-GlcNAc moiety. To gain insight into this important question, the crystallizable OGA_cryst_ construct (Figure 3a) was applied to obtain a series of structures in complex with distinct O-GlcNAcylated peptides, including one derived from the tumor suppressor p53 (Figure 3b) [69,75]. Notably, the peptide parts of these substrates are all bound in the clefts created by the dimerization of OGA, termed “substrate-binding clefts”, which consist of the top surface of the catalytic domain of one OGA monomer and the stalk domain of the sister monomer (Figure 3b). The O-GlcNAc binding conformation of these glycopeptides is highly conserved in the OGA’s catalytic pocket, supporting that O-GlcNAc is an indispensable element for OGA substrate recognition. However, differential interactions between the peptide side-chains and OGA cleft surface residues have been observed. Mutations of related binding residues on the glycopeptide or OGA cleft surface impaired their interaction, suggesting that OGA is capable of recognizing specific features of glycosylated substrates beyond the O-GlcNAc moiety. This hypothesis is in line with the quantitative proteomic analysis, which detected a broad range of O-GlcNAc half-lives on different protein substrates as responding to OGA activity in human leukemia Jurkat cells [76]. Taken together, these structural and proteomic results support that protein interactions with the OGA substrate-binding cleft do exist and can contribute to OGA substrate binding and discrimination.

Besides the helical bundle as shown in the crystal structure (Figure 3b), the OGA stalk domain also comprises an unstructured, disordered loop region (residues 401 aa–552 aa, predicted structure model of full-length OGA is shown in Figure 3c) [67]. It is known that IDR of a protein is one of the main interaction platforms for the assembly of multiprotein complexes and may play distinct roles depending on the biological context [45,77,78]. IDR often initiates high specificity but low affinity interactions due to the dramatic entropy change from disorder-to-order transition upon binding to a ligand/protein [45,77,78]. Therefore, IDR could be essential for the plasticity of OGA interactions with various proteins, which is also a feature of cancer-specific PPIs [45]. Although it remains largely unknown how OGA employs the disordered region in protein interactions, this region is important for OGT binding, in vitro O-GlcNAcylation, and the proteolytic cleavage of OGA [63,79]. These results suggest that the stalk domain orchestrates OGA–protein interactions through both ordered and disordered regions. Compared to other hexosaminidases, another unique feature of OGA is the C-terminal pHAT domain (Figure 3a,c). This domain was initially identified using SMART (Simple Modular Architecture Research Tool) [80] and showed modest sequence similarity to other known acetyltransferases [5]. However, the recombinantly purified pHAT domain or OGA alone cannot bind to acetyl-CoA; thus, the exact function of this domain remains elusive [81,82]. Since the pHAT domain is highly conserved in mammalian OGAs, it is reasonable to hypothesize that it coordinates with other domains of OGA to enhance the specificity and flexibility of OGA–protein interactions [5]. It is also possible that the pHAT domain modulates OGA–protein interactions through accessory proteins in a context-specific manner. Interestingly, studies of sOGA lacking the pHAT domain showed decreased glycosidase activity in vitro [83,84]. Additionally, sOGA regulates proteasome activity and global poly-ubiquitination in HeLa cells, implicating distinct PPIs as opposed to full-length OGA [84]. Furthermore, OGA with point mutations in the pHAT domain showed aberrant protein interactions compared to wild-type OGA (see “Systematic analyses of OGT/OGA associated PPI networks in cancer” section below) [18]. The unique structural features and related studies mentioned above support that OGA regions beyond the catalytic pocket play pivotal roles in regulating the specificity and plasticity of OGA–protein interactions. Future investigations on the binding modes of OGA with different protein substrates and non-substrate partners are expected to further reveal the molecular mechanisms underlying OGA–protein interactions and their roles in pathological processes.

## 3. Systematic Analyses of OGT/OGA Associated PPI Networks in Cancer

O-GlcNAc cycling enzymes (OGT and OGA) operate their functions by interactions with other biomolecules. The multiprotein complexes of OGT/OGA are of fundamental importance to decipher their roles in various biological processes. As previously reported that aberrant PPIs underlie the etiology of cancer, decoding the molecular connections of dysregulated OGT/OGA–protein networks in cancer will be important for therapeutic innovations [85]. To date, rapidly accumulating knowledge of O-GlcNAcylated proteins, and a few high-throughput studies of OGT interactions including the analyses using protein microarray in vitro [86] and the quantitative proteomics in mouse embryonic fibroblast (MEF) cells [87], have enabled the establishment of a massive compendium about the OGT interacting proteins (OGT-PIN) [88]. Less information about OGA binding partners has been disclosed; however, a potential high-level of overlap may exist between OGT– and OGA–substrate interactions. Despite these propitious findings, only a few systematic analyses of OGT/OGA-associated PPI networks have been reported in cancer models. Below, we highlight the representative studies and hope to encourage new investigations that can expand our current understanding from substrate-focused subnetworks to more comprehensive PPI networks of OGT/OGA. This system-level information will aid in the identification of cancer-specific PPIs and may inspire the rational design of innovative therapeutic strategies for cancer.

The profiling of OGT/OGA-associated PPIs in cancer cells typically apply affinity purification or proximity biotinylation coupled with quantitative LC-MS/MS analysis (AP-MS or BioID-MS) [89,90]. For instance, the functions of mOGT in breast cancer cells have been investigated through its interactomes [91]. Compared to ncOGT, the relatively short TPR region (9 instead of 13.5 TPRs, Figure 2a) and the unique mitochondrial localization imply that mOGT may form a PPI network different from ncOGT. This is in agreement with the distinct substrate profiles and cytotoxic effects of mOGT observed in mammalian cells [92,93]. Following endogenous ncOGT knockdown and HaloTag-mOGT affinity purification from mitochondrial fractions, more than 40 mitochondrial proteins have been identified as mOGT binding partners in at least two different breast cancer cell lines compared to HaloTag control [91]. These proteins participate in almost every aspect of mitochondrial functions, including mitochondrial transport, respiration, translation, fatty acid metabolism, apoptosis, and mtDNA processes. This finding is also in line with the observation in cervical cancer HeLa cells that mOGT contributes to mitochondrial structure and function, as well as cancer cell survival [94]. Surprisingly, a few nuclear proteins were also detected as mOGT binders. This implicates potentially distinct roles of different OGT isoforms in cancer cells. While these discoveries on mOGT–protein interactions are informative, further analyses will be needed to define cancer-specific PPIs of mOGT.

Protein interactions with O-GlcNAc cycling enzymes consist of transient or weak interactions. The recently developed proximity biotinylation (BioID) technique is well-suited for this type of detection [90]. It was applied to investigate OGA-mediated oxidative stress response in osteosarcoma U2OS cells [95]. In this study, ectopic expression of OGA fused with biotin ligase mBirA can biotinylate proteins bound or in proximity to OGA. The changes of OGA–protein interactions in response to H_2_O_2_-induced oxidative stress were identified by LC-MS/MS detection of biotinylated proteins. As a result, dozens of OGA binding partners have been identified as significantly regulated, including fatty acid synthase (FAS), filamin-A (FLNA), heat shock cognate 70-kDa protein (HSC70), and OGT. Interestingly, biochemical analyses further revealed that the interaction with FAS suppressed OGA’s catalytic activity and modulated the stress adaptation of cancer cells. Using the AP-MS approach, another study identified OGA–protein interactions in HeLa cells [18], showing significant enrichment of cellular functions, such as RNA splicing, mRNA processing, cytoskeleton organization, intracellular transport, and mitosis (GO term analysis of the data from Table S1 in [18] using DAVID [96,97]). Intriguingly, many of these OGA PPI functions were absent in the OGA pHAT domain mutant (Y891F), except for RNA splicing and mRNA processing (GO term analysis of the data from Table S2 in [18] using DAVID), suggesting that the pHAT domain is indispensable for maintaining the integrity of OGA PPI networks. Notably, the same study also found that OGA was upregulated in many types of cancer and drove aerobic glycolysis and tumor growth by inhibiting pyruvate kinase M2 (PKM2). Further experiments suggested that the activity of PKM2 was dysregulated by OGA complex-associated acetylation and O-GlcNAcylation under cancer-related high glucose conditions. Overall, these studies have begun to uncover the abnormal PPIs of OGT/OGA in cancer models. With advances in proteomics and bioinformatics, we envision that the systematic analyses of protein interactions with OGT/OGA (not restricted to O-GlcNAcylated proteins) will identify new, cancer-specific PPIs and help define the oncogenic properties of these O-GlcNAc cycling enzymes in cancer biology.

## 4. Dysregulated Protein Functions by Rewired OGT/OGA Protein Networks in Cancer

**Figure 4:**
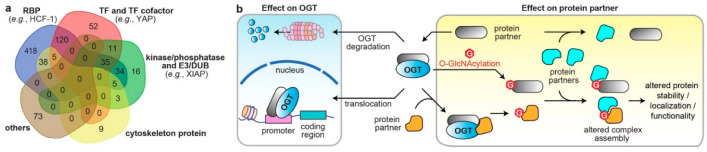
The diverse molecular impacts of protein interactions with O-GlcNAc cycling enzymes. (**a**) Classification of reported OGT/OGA binding partners in cancer. The information of OGT and OGA binding partners was from database OGT-PIN (high-stringency interaction proteins) and PINA, respectively. The binding partners were categorized using the databases AnimalTFDB 3.0 [98], KinMap [99], DEPOD [100], UbiBrowser 2.0 [101], UbiNet 2.0 [102], RBP2GO [103], and gene ontology (GO term: cytoskeleton) from DAVID [97]. The venn diagram was generated from http://bioinformatics.psb.ugent.be/webtools/Venn/ (accessed on 15 October 2022). RBP, RNA binding protein; TF, transcription factor; E3, E3 ubiquitin ligase; DUB, deubiquitinase. (**b**) Different molecular mechanisms underlying the protein interactions with O-GlcNAc cycling enzymes in dysregulating protein functions in cancer cells (OGT is shown as an example).

PPIs are the frameworks for signal transmission in conducting cellular events. The broad-spectrum effect of O-GlcNAcylation suggest that OGT/OGA PPIs regulate the spatiotemporal communication of many biological processes. Analysis of all reported interacting partners of human OGT/OGA in the curated databases, OGT-PIN (high-stringency partners) [88] and PINA [43], demonstrated diverse molecular characters, including nucleotide binder, kinase/phosphatase, E3 ubiquitin ligase/deubiquitinase (DUB), and cytoskeleton (Figure 4a). Herein, abnormal OGT/OGA networks can affect proteins at multiple levels, including PTM, conformation, and association with other biomolecules, which consequently modulate the enzyme activity, protein stability and transportation, among others [7,9]. Below, we highlight a few representative examples, in which the OGT/OGA–protein interactions have been validated by orthogonal methods, such as immunoprecipitation, to demonstrate the diverse molecular impacts of these PPIs on the malignant programming of cancer cells (Figure 4b). These studies illustrate that O-GlcNAc cycling enzymes can form divergent protein complexes with substrates and/or non-substrate partners and execute multifunctional roles in cancer. While most studies were focused on OGT, it is likely that OGA could apply similar mechanisms.

### 4.1. Altered Cellular Localization and Protein Stability of O-GlcNAc Cycling Enzymes

The chromatin association of OGT and OGA has been detected in different types of cells, which is consistent with their essential roles in transcription and epigenetic regulation [104,105]. Some binding partners/substrates of OGT have been found to assist the recruitment of OGT to promoter regions [105,106,107,108,109,110]. One of the most studied partners is mSin3A, an isoform of mammalian Sin3 that serves as a scaffold for histone deacetylase complexes in gene regulation [111]. In hepatoma HepG2 cells, mSin3A interacts with the OGT N-terminal TPR region and recruits it to the promoters for transcriptional repression [109]. This example demonstrates that PPIs modulate OGT translocalization and its associated epigenetic functions. On the other hand, aberrant protein interactions in cancer cells can alter the stability of O-GlcNAc cycling enzymes, which may further dysregulate their PPI networks and affect cancer cell growth. In a surprising discovery, histone demethylase LSD2 was found to act as an E3 ubiquitin ligase, to directly interact with OGT, and to induce its ubiquitin-dependent proteasomal degradation [112]. While the O-GlcNAcylation status of LSD2 remains unknown, the interaction of LSD2 and OGT displayed an anti-growth effect in lung cancer A549 cells by reducing the stability and protein level of OGT. Another OGT interactor with E3 ubiquitin ligase activity is the X-linked inhibitor of apoptosis protein (XIAP) [113]. In colon cancer HCT116 cells, XIAP directly interacts with OGT and induces its proteasomal degradation. More interestingly, XIAP can be O-GlcNAcylated, and the modification is essential for the E3 ubiquitin ligase activity of XIAP toward OGT specifically, but not other protein substrates in HCT116 cells. Thus, O-GlcNAcylated XIAP suppresses cancer cell growth and invasion by degrading OGT. However, significantly reduced OGT would downregulate O-GlcNAcylation of XIAP. This reciprocal modulation is an elegant example showing how cancer cells control the stability/level of O-GlcNAc cycling enzymes through specific protein interactions.

### 4.2. Effects on the Direct Binding Partners/Substrates of O-GlcNAc Cycling Enzymes

Aberrant interactions with O-GlcNAc cycling enzymes can modulate the O-GlcNAcylation of binding partners, leading to altered protein stability, localization, and functionality [7,12]. This is a prevailing mechanism of the abnormal OGT/OGA networks underlying cancer development, also in line with the fact that O-GlcNAcylation is essential for transcription and translation, which are often reprogramed in cancer [114,115]. As previously reported, O-GlcNAcylation of many transcription factors, such as Sp1 [116], FOXO1 [117], p53 [118], and NF-κB [119], can upregulate their activities in cancer cells by increasing their protein stability and nuclear translocation. The molecular mechanisms usually involve aberrant OGT–protein-association and the resulted O-GlcNAcylation that may alter the assembly of multiprotein complexes (Figure 4b). A typical example is the notorious cancer promoter, SIRT7, which is a member of the NAD^+^-dependent deacetylase Sirtuin family [120]. OGT was detected in complex with SIRT7 in different pancreatic cancer cell lines [121]. The interaction occurred through the C-terminus of SIRT7 and the TPR region of OGT, inducing O-GlcNAcylation and facilitating the stabilization of SIRT7 by interfering its interaction with REGγ proteasome. Elevated SIRT7 deacetylated the lysine 18 of histone H3 (H3K18), promoted the enrichment of SIRT7 at the promoters, and inhibited the expression of tumor suppressors to fuel cancer progression. In another example, the ribosomal receptor for activated C-kinase 1 (RACK1), an important component of the 40S ribosome subunit, was identified to interact with OGT in hepatoma cells [122]. O-GlcNAcylated RACK1 showed significantly higher stability and ribosome localization, and more importantly, promoted its association with another kinase PKCβII, which is an essential signaling molecule for RACK1 initiated translation. The RACK1/PKCβII complex stimulated the translation and expression of several oncogenes, driving hepatocellular carcinogenesis. Rewired protein networks of OGT have also been reported for the oncogene YAP, which is a transcription factor in Hippo signaling [123]. Abnormally activated Hippo pathway and YAP-stimulated gene expression give rise to uncontrolled cell growth and tumor formation [124]. In contrast, YAP phosphorylation by kinase LATS1 increases its cytoplasmic translocalization and degradation, leading to negative regulation of Hippo signaling [124,125]. Interestingly, YAP was found to interact with OGT in vitro and in cells, and the O-GlcNAcylation on the S109 residue suppressed the association of YAP with LATS1, and the phosphorylation of YAP at S237. Hence, in pancreatic cells, the aberrant OGT interaction and O-GlcNAcylation of YAP promote its dephosphorylation, nuclear localization, and transcriptional activity, fueling Hippo signaling and tumor growth [123]. Cancer cells also apply similar means to antagonize the genotoxicity from commonly used chemotherapeutic agents, such as adriamycin (adm) [126]. A study in breast cancer cells detected that MTA1, a highly deregulated oncogene involved in the stress adaptation of cancer, is an OGT substrate [127,128]. Intriguingly, MTA1 displayed enhanced interaction with OGT in adm-resistant (MCF7/ADR) cells compared to adm-sensitive (MCF7) cells [128]. Immunoprecipitation and genome-wide analysis further showed that O-GlcNAcylation of MTA1 promoted its association with components of nucleosome remodeling and histone deacetylation (NuRD) complex, including HDAC1, MBD3, and CHD4, and recruited MTA1 to the promoter of stress-adaptive genes for transcriptional activation. Therefore, MTA1–OGT interaction and its aberrant O-GlcNAcylation protected the breast cancer cells against genotoxic stress, leading to drug resistance. It would be interesting to evaluate whether OGA–protein interactions also engage in modulating similar protein complexes in cancer. In summary, aberrant OGT–protein interactions and O-GlcNAcylation deregulate the assembly of the protein with other biomolecules, leading to diminished proteolysis and upregulated functions that may promote cancer cell growth. However, exceptions do exist in other cellular conditions [129]. Please refer to [11,13,31] for more detailed reviews about the regulation of O-GlcNAcylation on protein substrates in cancer.

### 4.3. Modulations through Binding Adaptors

One of the hypotheses regarding how OGT/OGA recognizes their protein substrates is through protein adaptors [56]. Recently, a few OGT adaptors have been identified in different types of cancer cells. In hepatoma FAO cells, it was reported that the interaction of OGT with HCF-1, a transcriptional cofactor playing critical roles in cell cycle and stem cell regulation, enhanced the O-GlcNAcylation of transcription factor PGC-1α [130]. Further analysis demonstrated that O-GlcNAcylated PGC-1α displayed increased stability by forming a stronger complex with deubiquitinase BAP1, and thereby promoted the gluconeogenic gene expression in response to glucose availability. Another study discovered that an anti-viral and pro-inflammatory protein, IFIT3, assisted OGT interaction with substrate VDAC2 in pancreatic adenocarcinoma and patient-derived primary cells [131]. VDAC2 is a channel protein involved in mitochondria-associated apoptosis [132]. Upregulated O-GlcNAcylation of VDAC2 protected highly metastatic cancer cells from chemotherapy-induced apoptosis, leading to drug resistance [131]. CEMIP, a cell-migration inducing protein promoting metastasis through glutamine metabolic reprogramming, is another OGT adaptor identified in colorectal cancer and was recently discovered as a metastasis-related protein [133]. It stabilized the interaction of OGT with substrate β-catenin, resulting in elevated O-GlcNAcylation of β-catenin and displacing it from its complex with cadherins in the cytomembrane for nuclear translocation and transcription regulation. CEMIP- and OGT-induced nuclear accumulation of β-catenin can transactivate genes in metabolic reprogramming and promote tumor growth. While the O-GlcNAcylation status of CEMIP was not reported in this case, its middle and C-terminal regions were responsible for OGT and β-catenin binding, respectively. Intriguingly, cells with CEMIP knockdown or overexpression altered the O-GlcNAcylation of many proteins, suggesting that this adaptor mediates the interactions of O-GlcNAc cycling enzymes with many other substrates. Overall, these interesting discoveries strongly support that the binding adaptors can modulate the PPI networks of O-GlcNAc cycling enzymes in malignant transformation. Future investigations are expected to uncover additional novel adaptors and their mechanisms of action in cancer.

## 5. Future Perspectives

**Figure 5:**
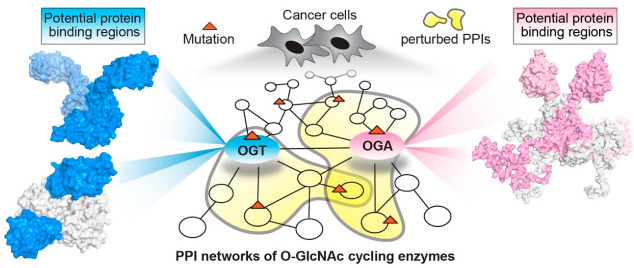
Integrating complementary approaches holds the promise of identifying key players (e.g., pathogenic mutations, altered pathways, or protein interactions) that drive cancer malignancies and will facilitate therapeutic innovations.

Cancer is typically defined as a genetic disease driven by dysregulated gene expression and/or oncogenic mutations [134]. Cancer research has primarily focused on a few frequently mutated genes, such as the tumor suppressor p53, while the vast majority remain largely unexplored but hold potential as new targets for drug development [135]. In this context, OGT and OGA are of special interest considering their fundamental roles in cells and their dysfunctions in different types of cancer, despite less studies on OGA [11]. However, genetic knockdown or chemical inhibition of their active sites may induce undesired side effects given their essential functionality [34,35,36,37]. Hence, new strategies that can target their functions specifically toward a subset of proteins without perturbing global O-GlcNAc homeostasis would be highly desirable. These O-GlcNAc cycling enzymes are potentially highly connected “hubs” in PPI networks, explaining their multifunctional roles in the cell. Interestingly, recent studies on a broad panel of disease-related proteins have found that many disease-driven proteins are “hub” proteins [46,136]. More importantly, a significant fraction (~33%) of their missense mutations affect only specific protein interactions rather than their protein folding/stability or global functions [136]. Hence, targeting specific protein interactions of OGT/OGA by PPI modulators (e.g., PPI inhibitors) is a promising new area for drug development. PPIs are critical networks underling all cellular events and possess system-level information to help decipher the molecular basis of diseases. In the past decade, the substrate interactions of O-GlcNAc cycling enzymes in several cancer models have become available, but the information for comprehensive OGT/OGA PPIs, including non-substrates, is far from being completed for many types of cancer. For the future development of specific OGT/OGA PPI modulators to combat cancer, significant progress in several key areas will be instrumental. For instance, high quality, extensive, and systematic profiling of OGT/OGA protein networks in a variety of cancer models and clinical samples will be essential to identify and extract functional modules or subsets of networks from the vast information to establish molecular connections with clinical phenotypes. On the other hand, protein complex structures will provide invaluable atomic details to guide rational design of PPI modulators. Unfortunately, obtaining high-resolution structures of full-length OGT, OGA, and their protein complexes remains a challenge. As mentioned above, their protein interactions may consist of weak or dynamic multiprotein associations, which could be difficult to determine using a single traditional structural method. However, integrative structural biology that synergizes multiple complementary experimental and computational methods would be a powerful strategy for future characterization of OGT/OGA–protein complex structures. In addition, advances in computational modeling offer an alternative approach to predict protein–protein interface and hotspot residues [39]. Interestingly, a bioinformatic study showed that mutations located at PPI interfaces are highly correlated with cancer patient survival [137]. According to public cancer databases, hundreds of OGT/OGA mutations, and a lot more mutations of their protein partners, have been identified through next-generation sequencing [138,139]. However, the functional impacts of a majority of these mutations remain uncharacterized. Given the functional diversity of OGT/OGA, it is likely that different mutations within the same gene may produce distinct interaction profiles, leading to differential cancer phenotypes or responses to the same anti-cancer treatment. Integrating complementary approaches at the systems level (e.g., genome, transcriptome, proteome, interactome) and molecular level (e.g., protein complex structure characterization, functional and mechanistic analyses of purified components) holds the promise of identifying key players (e.g., pathogenic mutations, altered pathways, or interactions) that drive most of the characteristic malignancy (Figure 5). The synergy of these complementary approaches will produce critical insights into the genotype and phenotype relationship, and will facilitate new diagnosis, prognosis, and therapeutic interventions in the context of precision medicine.

## 6. Conclusions

O-GlcNAc cycling enzymes (OGT and OGA) regulate the functions of proteins in a broad range of cellular processes and their dysfunctions have been detected in many cancer types. In this review, we highlighted recent progress on the structural characterization of OGT and OGA, as well as their emerging roles in protein interaction networks in several cancer models. We also discussed the potential of leveraging integrated approaches to dissect the molecular connections of OGT/OGA dysfunction and cancer phenotypes and manipulating their protein interactions as novel targets for anti-cancer therapeutic interventions.
